# Spot Weight Adaptation for Moving Target in Spot Scanning Proton Therapy

**DOI:** 10.3389/fonc.2015.00119

**Published:** 2015-05-28

**Authors:** Paul Morel, Xiaodong Wu, Guillaume Blin, Stéphane Vialette, Ryan Flynn, Daniel Hyer, Dongxu Wang

**Affiliations:** ^1^Laboratoire Informatique Gaspard Monge (LIGM), UMR CNRS 8049, Université Paris-Est, Paris, France; ^2^Department of Radiation Oncology, The University of Iowa, Iowa City, IA, USA; ^3^Department of Electrical and Computer Engineering, The University of Iowa, Iowa City, IA, USA; ^4^UMR 5800, Laboratoire Bordelais de Recherche en Informatique (LaBRI), Université de Bordeaux, Talence, France

**Keywords:** intensity-modulated proton therapy, interplay effect, spot scanning, spot weight adaptation, motion

## Abstract

**Purpose:**

This study describes a real-time spot weight adaptation method in spot-scanning proton therapy for moving target or moving patient, so that the resultant dose distribution closely matches the planned dose distribution.

**Materials and methods:**

The method proposed in this study adapts the weight (MU) of the delivering pencil beam to that of the target spot; it will actually hit during patient/target motion. The target spot that a certain delivering pencil beam may hit relies on patient monitoring and/or motion modeling using four-dimensional (4D) CT. After the adapted delivery, the required total weight [Monitor Unit (MU)] for this target spot is then subtracted from the planned value. With continuous patient motion and continuous spot scanning, the planned doses to all target spots will eventually be all fulfilled. In a proof-of-principle test, a lung case was presented with realistic temporal and motion parameters; the resultant dose distribution using spot weight adaptation was compared to that without using this method. The impact of the real-time patient/target position tracking or prediction was also investigated.

**Results:**

For moderate motion (i.e., mean amplitude 0.5 cm), D95% to the planning target volume (PTV) was only 81.5% of the prescription (R_X_) dose; with spot weight adaptation PTV D95% achieves 97.7% R_X_. For large motion amplitude (i.e., 1.5 cm), without spot weight adaptation PTV D95% is only 42.9% of R_X_; with spot weight adaptation, PTV D95% achieves 97.7% R_X_. Larger errors in patient/target position tracking or prediction led to worse final target coverage; an error of 3 mm or smaller in patient/target position tracking is preferred.

**Conclusion:**

The proposed spot weight adaptation method was able to deliver the planned dose distribution and maintain target coverage when patient motion was involved. The successful implementation of this method would rely on accurate monitoring or prediction of patient/target motion.

## Introduction

Spot scanning proton therapy has been shown to be a dosimetrically superior radiation delivery modality in cancer treatment (1). The ability to accurately place proton beam’s Bragg peak inside a target is crucial to the realization of proton beam’s dosimetric benefits. Yet multiple sources of uncertainty exist in the process of treatment planning and treatment delivery (2–4), weakening the potential advantage of spot scanning proton therapy. Among these uncertainties, intra-fraction motion, especially the respiratory motion, is of particular concern (5), due to its less predictable nature.

Motion management and motion mitigation strategies have been proposed for spot scanning proton and particle beam therapy. One of the most common techniques, as used in photon radiation as well, is the beam gating (6, 7), which consists of irradiating the patient only when the patient reaches a certain phase of the breathing cycle. During gating, breathing can also be regulated through coaching by audio or visual feedback in order to limit the cycle’s variability (6). Motion may also be suppressed by breath-hold or active breathing control techniques (8). Functional apnea or mechanical ventilation may be used under anesthesia (9). These techniques are promising solutions but tend to be constraining for the patient.

Other strategies not constraining the patient have been investigated. Repainting (also called re-scanning) (10) consists of delivering the planned dose into several cycles instead of a single pass. The goal is to obtain a high scanning speed by reducing the time spent at each spot position and reduce the risk of large localized dose discrepancies. While this technique is encouraging, it does not take into account the patient motions during the delivery. Four-dimensional (4D) treatment planning (11) using acquired patient motion information to aid treatment planning is also proposed. Beam tracking method (12, 13) calculates the target displacement and uses this information to adapt the beam position and beam energy to aim the planned target point in the patient. This method is particularly promising; however, it requires the adjustment of both the position and the energy of the delivering pencil beam. A number of reviews and reports (5, 14, 15) provide good summaries of the existing investigative or implemented methods.

In this work, we propose an alternative technique that only adapts the pencil beam (spot) weights to account for the patient intra-fraction motion. Like the beam tracking method, this method too relies on the real-time information of target spot location, but it allows the scanning magnets to change the beam position and energy following a planned and quasi-continuous trajectory, instead of constantly chasing the target spot. The response time in this method is, therefore, potentially shorter; especially considering that energy changes in beam tracking method may be the bottleneck of effectively tracking a target.

## Materials and Methods

### The principle of spot weight adaptation

The overall principle of the spot weight adaptation strategy is introduced here. At a given moment, the weight of a pencil beam at its current energy and direction is adapted to the weight planned for the target spot that is under the irradiation of the current beam at this moment. The weight can be obtained from the patient and tumor displacement at voxel levels, as well as the weight of the pencil beam that was originally planned for this target spot.

**Figure 1:**
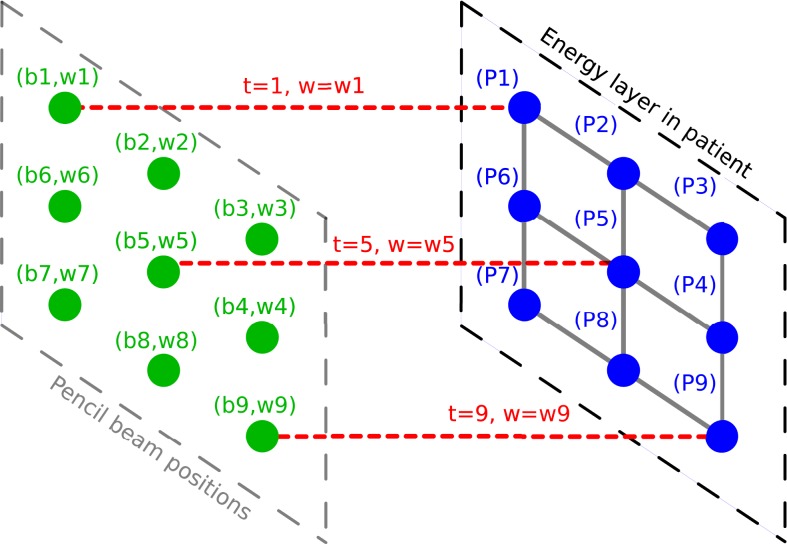
**Illustration of the planned spot delivery for a static patient**. Delivering spot positions are represented in green. Pencil beams are represented in red. Target spots in the patient are represented in blue.

More formally, let us consider a treatment plan, for which the beam of one energy layer is represented by the points (*b*_*i*_, *w*_*i*_), where *i* indicates the beam index, *b*_*i*_ the beam position and *w*_*i*_ the corresponding planned spot weight. This plan induces the irradiation of a set of respective target spot locations (*P*_*i*_) in the static patient, located at the depth of the Bragg peak. For the purpose of implementing spot weight adaptation only, we define (*P*_*i*_) as irradiated by the delivering spot (*b*_*i*_, *w*_*i*_). The total dose to a certain target spot (*P*_*i*_) will include contributions from other delivering spots and other energy layers. In a treatment plan generated for a static patient, the delivering spots (*b*_*i*_, *w*_*i*_) and target spots (*P*_*i*_) have a one-to-one relationship, and therefore often not considered separately (Figure 1).

**Figure 2:**
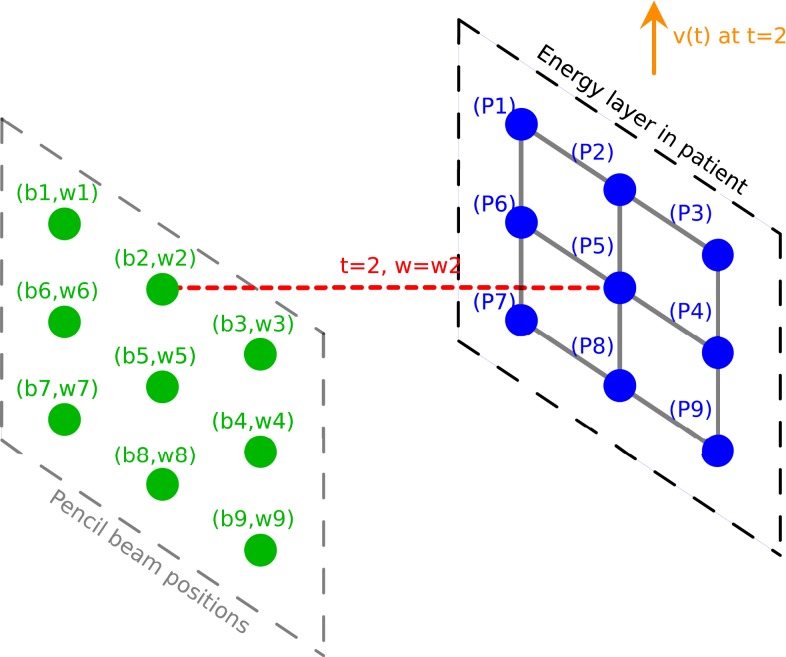
**Illustration of the delivery for a moving patient without spot weight adaptation**. Delivering spot positions are represented in green. Pencil beams are represented in red. Target spots in the patient are represented in blue. The 2D displacement vector is represented in orange.

**Figure 3:**
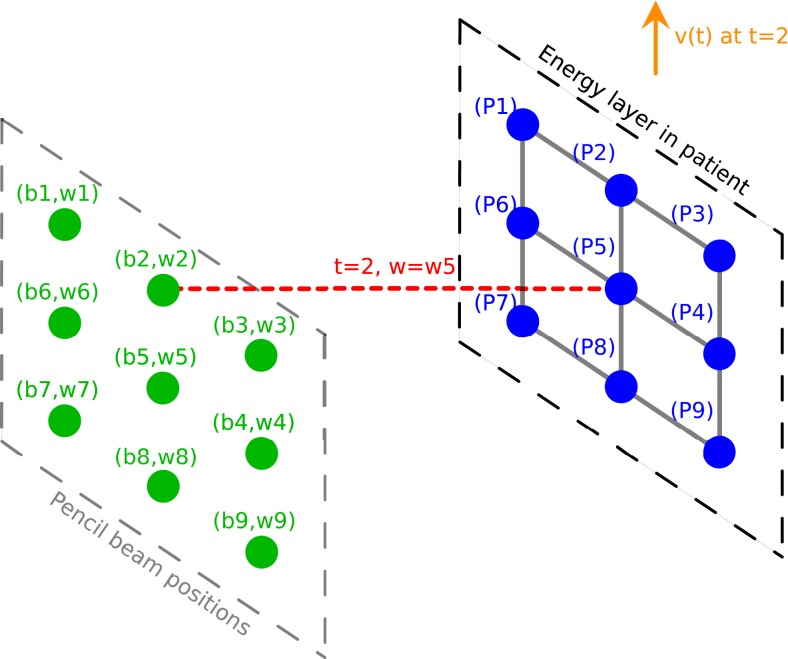
**Illustration of the delivery for a moving patient with the spot weight adaptation; the weight now is w5**. Delivering spot positions are represented in green. Pencil beams are represented in red. Target spots in the patient are represented in blue. The 2D displacement vector is represented in orange.

During the treatment delivery for a static patient, the proton machine changes energy and spot position, and deliver the planned set of pencil beams (*b*_*i*_, *w*_*i*_) one spot at a time. If the target spots are now moving relative to the delivering spots, the delivering spot may no longer irradiate the intended target spot. However, the planned dose distribution may be achieved if the target spot *P*_*i*_ receives the planned beam weight, *w*_*i*_, in terms of Monitor Unit (MU), even if it is from a different delivering spot *b*_*j*_. A simple illustration of this method is shown in Figure 2. The motion is considered in 2D, in the plane orthogonal to the beam direction, as a displacement vector v(*t*). As an example, at time *t* = 2, the beam will be at position *b*_2_ and will deliver a weight *w*_2_, following the sequence of control points in the treatment control system as defined by the plan. However, at this moment another target point *P*_5_ would be irradiated instead of the planned target spot *P*_2_, and *P*_5_ would receive an irradiation corresponding to *w*_2_ instead of its planned value *w*_5_. The spot weight adaptation technique relies on the adaptation of the beam weight for delivering spot *b*_2_, which is set to *w*_5_ instead of *w*_2_ (see Figure 3).

### Implementation of spot weight adaptation

Algorithm 1 represents the overall process of the delivery with adapted spot weights.

**Algorithm 1:** **Algorithm of the compensated delivery**. TP is the treatment plan provided by the user. *l*_pos_ is the list of all delivering spots within a certain energy layer, including those with zero weights based on the plan. *l*_plan_ is the list of the delivering spots with non-zero spots.

**Figure 4:**
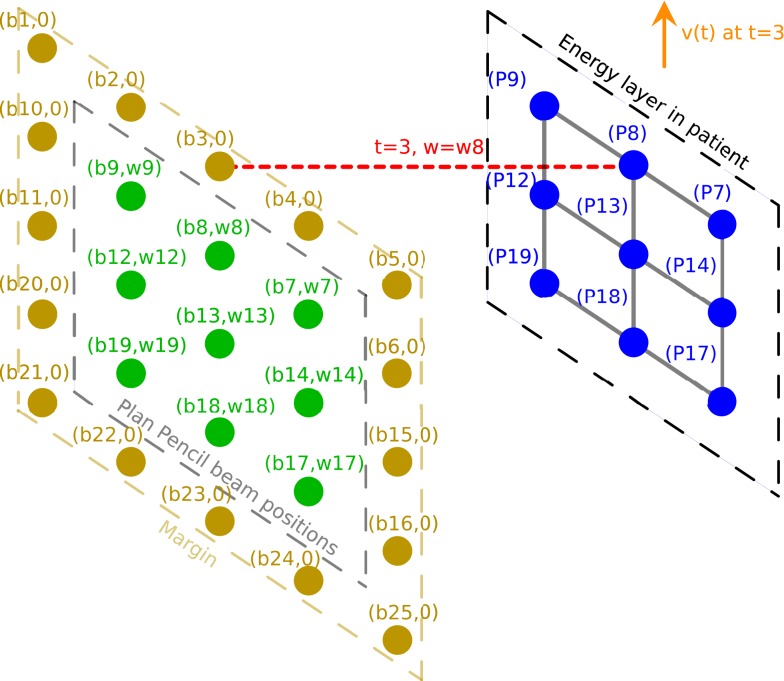
**Illustration of the delivery for a moving patient with spot weight adaptation where a margin is added around the planned delivering spots**. Planned delivering spots are represented in green. Beams are represented in red. Target spots in the patient are represented in blue. The patient displacement vector is represented in orange.

**begin**
**foreach** Gantry angle **do**
**foreach** Energy level **do**
Build *l*_pos_ the list of all the delivering spots;
//Note: if margin is used, include in *l*_pos_ the margin beam positions with
weight = 0 if margin is used. See description later about margin.
Build *l*_plan_ the list of all the delivering spots with weights >0;
**while** *l*_plan_ is not empty **do**
**foreach** Delivering spot *b*_*i*_ in *l*_pos_ **do**
Get the patient displacement vector *v*(*t*) and target spot (*b*_*k*_, *w*_*k*_) if available
//Obtained from the motion monitoring system and/or motion
modeling; see Motion Information section below.
//*w*_*k*_ being the weight associated to *b*_*k*_ in treatment plan
Irradiate the target spot with (*b*_*i*_, *w*_*k*_) or part of *w*_*k*_;
Update *l*_plan_: remove *w*_*k*_ or part of *w*_*k*_ from *b*_*k*_;
return;

For each field with *l*_pos_ is the list of all delivering spots within a certain energy layer, and the list *l*_plan_ is built with all the delivering spots with non-zero spots. The pencil beam moves at the planned positions in *l*_plan_ in order. At time *t* the beam is at position *b*_*i*_, and its weight is adapted to value *w*_*k*_ to reflect the new target spot *b*_*k*_. MUs corresponding to entire or part of *w*_*k*_ may be delivered by *b*_*i*_, and *w*_*k*_ is updated to become its remaining weight or zero for *b*_*k*_. The beam then moves to the next position. To fully cover the target spots, Algorithm 1 needs to be repeated multiple times, i.e., re-scanning is necessary; otherwise the planned target spots may not be fully delivered. The number of re-scans can be reduced by adding a margin around the planned spot positions (see Figure 4). The size of the margin can be adjusted by user to its optimal value, which may depend on the extent and speed of patient motion.

### Motion information

Implementing the spot weight adaptation algorithm requires knowing or predicting the patient and target position *P*_i_(*t*) at each moment of beam delivery. This is the same process as what is performed in the beam tracking method (12). In practice, this can be achieved with reasonable accuracy in two ways: (1) by modeling patient motion through 4DCT acquired before treatment planning and then relating patient motion to respiratory phase observables during treatment; (2) or by monitoring patient motion during treatment in real time through devices such as Calypso^®^ system (Varian Medical Systems, Palo Alto, CA, USA). The spot weight adaptation method itself does not rely on any specific method to supply patient motion information. In this work for the purpose of proof-of-principle testing, a motion model is created for the testing case patient, and a certain patient monitoring system is assumed to monitor patient motion in real-time, with realistic time delay and measurement noise included.

### Motion simulation and dose computation

Motion simulation and dose computation were performed in in-house software named Motion Simulator for Proton Therapy (MSPT) (http://code.google.com/p/mspt/). MSPT is an open-source treatment planning software that enables 4D dose computation. MSPT can import patient CT, structure, and spot scanning treatment plan in DICOM RT format. It can simulate user-defined motion pattern on the patient dataset on a voxel-by-voxel basis, and compute delivered dose in each temporal moment considering the temporal model for the beam delivery system, using analytical dose computation algorithm. It then sums the dose for each patient voxel along the time axis and produces the total dose distribution for the patient. A detailed description about MSPT and its validation has been published elsewhere (Morel et al. under review). The weight adaptation algorithm was implemented in MSPT for the test casing described below.

### A testing case

**Figure 5:**
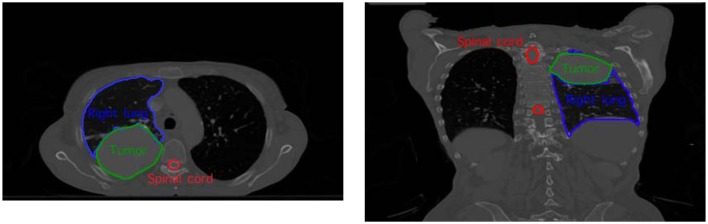
**The SBRT lung testing case with the delineation of the tumor and the organ-at-risk**.

To evaluate the compensation method, we perform motion simulation and dose computation on a SBRT lung case shown in Figure 5. Motions of moderate amplitude (0.5 cm in any dimension) and large amplitude (1.5 cm in any dimension) are simulated respectively. Different levels of error for motion monitoring are also introduced. The simulation settings are summarized in Table 1.

**Table 1:** **Motion and monitoring settings in different tests for the testing case**.

Test No.	Intra-fraction motion	Motion				Spot weight adaptation?	Motion monitoring error [σ_x_, σ_y_, σ_z_] in cm
		Mean amplitude [*x*, *y*, *z*] in cm	Amplitude Gaussian noise [σ_x_, σ_y_, σ_z_] in cm	Mean cycle period [*x*, *y*, *z*] in s	Cycle period Gaussian noise [σ_x_, σ_y_, σ_z_] in s	
1	No	–	–	–	–	–	–
2	Yes	[0.5, 0.5, 0]	[0.1,0.1, 0]	[4, 4, 0]	[0.8, 0.8, 0]	No	–
3	Yes	[1.5, 1.5, 0]	[0.3, 0.3, 0]	[4, 4, 0]	[0.8, 0.8, 0]	No	–
4	Yes	[0.5, 0.5, 0]	[0.1,0.1, 0]	[4, 4, 0]	[0.8, 0.8, 0]	Yes	[0, 0, 0]
5	Yes	[1.5, 1.5, 0]	[0.3, 0.3, 0]	[4, 4, 0]	[0.8, 0.8, 0]	Yes	[0, 0, 0]
6	Yes	[1.5, 1.5, 0]	[0.3, 0.3, 0]	[4, 4, 0]	[0.8, 0.8, 0]	Yes	[0.3, 0.3, 0]
7	Yes	[1.5, 1.5, 0]	[0.3, 0.3, 0]	[4, 4, 0]	[0.8, 0.8, 0]	Yes	[0.75, 0.75, 0]
8	Yes	[1.5, 1.5, 0]	[0.3, 0.3, 0]	[4, 4, 0]	[0.8, 0.8, 0]	Yes	[1.5, 1.5, 0]

*It is assumed that patient moves in sinusoidal patterns in *X* and *Y* axes independently (*X*–*Y* plane is perpendicular to the posterior beam’s direction), with Gaussian noise existing for mean amplitudes and cycle periods. Noise is assumed to be 20% of the mean value (16). It is further assumed that a real-time patient monitoring system exists, but with Gaussian error in its detected signals. Test 1 is the static plan; Test 2 is the test with moderate motion without spot weight adaptation, and Test 4 is the test with moderate motion with spot weight adaptation. Test 3 is the test with large motion but no spot weight adaptation; Tests 5–8 are tests with large motion and with spot weight adaptation but different levels of error in motion monitoring*.

Dose distribution color map and dose volume histogram (DVH) are used in evaluating the delivered dose distributions in different tests. To evaluate the quality of the delivered dose distribution, we consider the following criterion to evaluate whether the desired tumor coverage is maintained with patient motion: if 95% of the tumor volume receives 95% of the prescribed dose R_x_ (D95% ≥95% R_x_) in one fraction, then we consider target coverage is maintained in this fraction. In our situation the dose distribution used as a reference is the planned static delivery. Therefore, we will assume that the dose prescription R_x_ is the tumor D95% in the planned static dose distribution.

Patient imaging data used in this study are from consented patients and approved by the Institutional Review Board (IRB) at the University of Iowa (IRB #201211805).

## Results

### Spot weight adaptation for moderate amplitude motions

**Figure 6:**
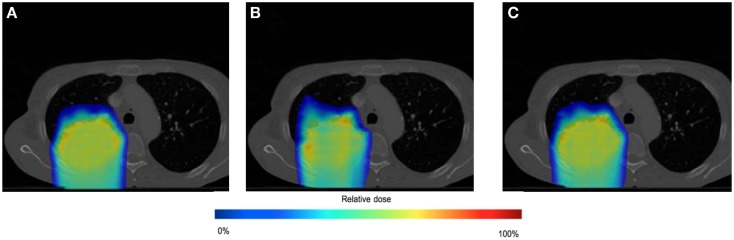
**Dose distributions for testing case in static situation [(A), Test 1], in moderate moving situation without spot weight adaptation [(B), Test 2], and in moderate moving situation with spot weight adaptation [(C), Test 4]**. Dose color map is relative to max dose in the static situation.

**Figure 7:**
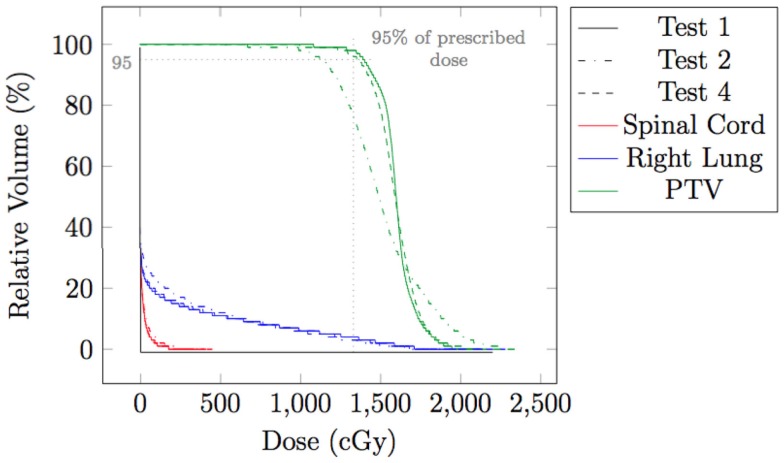
**Dose volume histogram (DVH) for testing case in static situation (Test 1), in moderate moving situation without spot weight adaptation (Test 2), and in moderate moving situation with spot weight adaptation (Test 4)**. 95% of R_X_ dose and 95% of planning target volume (PTV) are marked.

The effect of spot weight adaptation for a moderate motion amplitude (i.e., 0.5 cm) can be seen from the comparison between Tests 1, 2, and 4 (see Table 1 for test settings). Figure 6 shows the dose distribution color map of the three tests obtained near the center of the tumor. Figure 7 shows the DVH for the three tests.

From Figure 6, we can observe that with moderate motion (i.e., mean amplitude 0.5 cm) the dose distributions of the static and the spot-weight-adapted delivery are close, whereas dose heterogeneities are visible in the tumor in the case of the non-adapted delivery (Test 2, Figure 6B). Based on the DVH in Figure 7, PTV coverage is degraded in Test 2 without spot weight adaptation, where PTV D_95%_ is only 81.5% of R_X_; but with spot weight adaptation, PTV D_95%_ achieves 97.7%R_X_. The hot spot in the non-adapted Test 2 is also higher than the planned value. OAR dose in the non-adapted Test 2 is also higher, though not significantly worse than planned value.

### Spot weight adaptation for large amplitude motions

**Figure 8:**
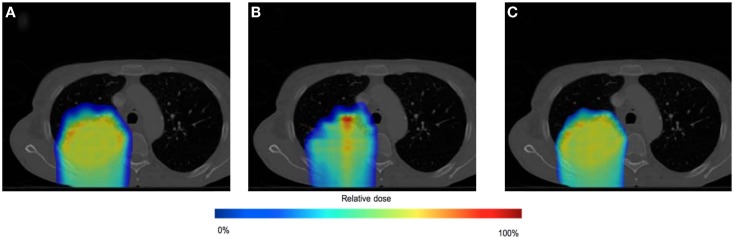
**Dose distributions for testing case in static situation [(A), Test 1], in large moving situation without spot weight adaptation [(B), Test 3], and in large moving situation with spot weight adaptation [(C), Test 5]**. Dose color map is relative to max dose in the static situation.

**Figure 9:**
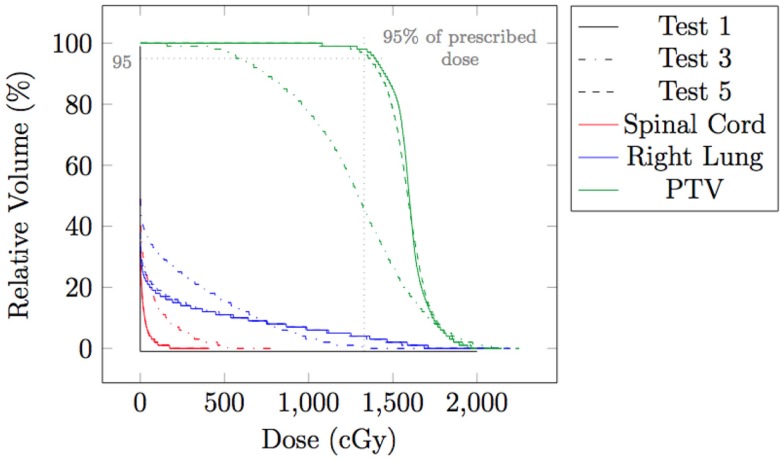
**Dose volume histogram (DVH) for testing case in static situation (Test 1), in large moving situation without spot weight adaptation (Test 3), and in moving situation with spot weight adaptation (Test 5)**. 95% of R_X_ dose and 95% of planning target volume (PTV) are marked.

The effect of the spot weight adapted delivery for a large motion amplitude (i.e., 1.5 cm) can be seen from the comparison between Test 1, 3, and 5 (see Table 1 for the settings). Figure 8 shows the dose distribution color map of the three tests obtained near the center of the tumor. Figure 9 shows the DVH for the three tests.

From Figure 8, we can observe that with large motion (i.e., mean amplitude 1.5 cm) the dose distributions of the static and the spot-weight-adapted delivery are close, whereas dose heterogeneities are very obvious in the tumor in the case of the non-adapted delivery (Test 3, Figure 8B). Based on the DVH in Figure 9, PTV coverage is degraded in Test 3 without spot weight adaptation, where PTV D_95%_ is only 42.9% of R_X_; but with spot weight adaptation, PTV D_95%_ achieves 97.7%R_X_. With spot weight adaptation, the delivered dose to OARs significantly deteriorated from the planned values; while with spot weight adaptation, delivered dose is very close to planned values, as shown from the DVH in Figure 9.

### The impact of the monitoring system accuracy

**Figure 10:**
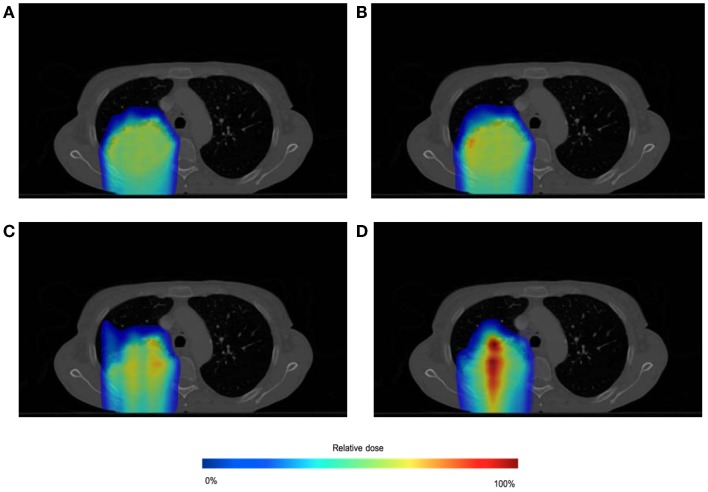
**Dose distributions for spot-weighted-adapted delivery with large motion, with different patient monitoring errors: σ = 0 cm (A), 0.3 cm (B), 0.75 cm (C), 1.5 cm (D)**. Dose color map is relative to max dose in the static situation.

**Figure 11:**
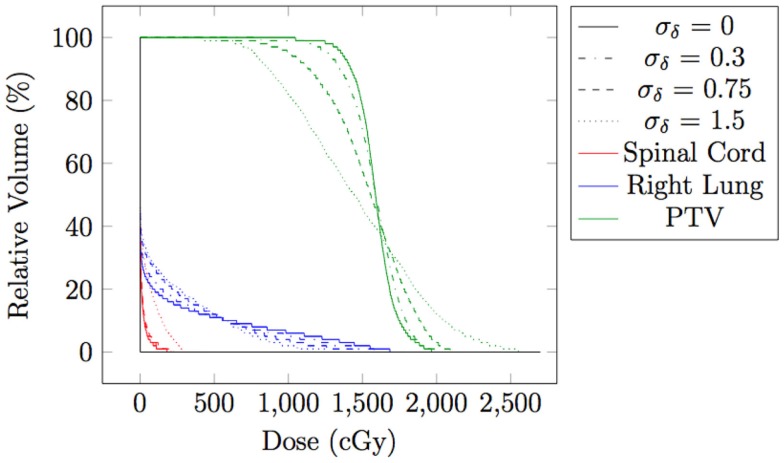
**Dose volume histogram for spot-weighted-adapted delivery with large motion, with different levels of patient monitoring errors**.

In the evaluations for spot weight adaptation with moderate and large motion (Test 2–5), an ideal patient monitoring system is assumed. In reality, patient motion information as an input to the spot weight adaptation algorithm should always contain errors. While both spatial and temporal errors can be present, only the impact of spatial errors is studied here, because the temporal error in detecting a current position of target spot and the delay of beam delivering can still be reflected in the error of target spot’s spatial location. Gaussian errors of three different levels of standard deviations were added: Tests 5 (σ = 0 cm), 6 (σ = 0.3 cm), 7 (σ = 0.75 cm), and 8 (σ = 1.5 cm). Table 1 has the summary of the settings for these tests. Figure 10 shows the dose distributions for Tests 5–8, and Figure 11 shows the DVH for Tests 5–8.

From both Figures 10 and 11, we can see that the accuracy of the monitoring system has an impact of the quality of the final dose distribution. PTV D_95%_ for Gaussian error of 0.3, 0.75, and 1.5 cm are 91.2% R_X_, 73.1% R_X_, and 55.7% R_X_, respectively.

## Discussion and Conclusion

When moderate (mean amplitude 0.5 cm) or large motion (mean amplitude 1.5 cm) is present in the testing case, delivering the plan without any adaptation deteriorated PTV coverage (Test 2 and Test 3, with PTV D_95%_ at only 81.5% R_X_ and 42.9% R_X_, respectively). This study proposed a method to adapt spot weight during delivery, with patient motion information as input. With spot weight adaptation, PTV coverage was able to achieve 97.7%R_X_ for moderate and large motion (Test 3 and Test 5), which is clinically acceptable.

The proof-of-principle example in this study did not include change in radiological depth, which would require spot weight adaptation across energy layers. The spot weight adaptation method in principle can adapt weight across energy layers, i.e., the method will look for the correct weight if the energy layer is different. Similar to the lateral spot margins as shown in Figure 4, an energy margin with spots of zero initial weight can be added. The zero-weight spots are to allow the delivering beam to move to that energy/place first, and then choose its MU based on the currently target spot. Re-scanning will be necessary in order to cover all spots and all energy layers. The number of re-scanning is likely smaller when the margin is larger. Since energy layer switch is usually in the order of seconds for spot scanning proton therapy, the number of re-scanning is important in determining the total treatment time. The relation between the number of re-scans and the size of the energy and lateral margins is currently being investigated and will be reported in future.

While the results also show the impact of the patient motion modeling and monitoring accuracy in spot weight adaptation method, just as in beam tracking method (12), this study shows that the spot weight adaptation itself is a promising strategy in mitigating the impact of patient motion during spot scanning proton therapy.

## Conflict of Interest Statement

The authors declare that the research was conducted in the absence of any commercial or financial relationships that could be construed as a potential conflict of interest.
